# Solving an MHC allele–specific bias in the reported immunopeptidome

**DOI:** 10.1172/jci.insight.141264

**Published:** 2020-10-02

**Authors:** Martin G. Klatt, Kyeara N. Mack, Yang Bai, Zita E. H. Aretz, Levy I. Nathan, Sung Soo Mun, Tao Dao, David A. Scheinberg

**Affiliations:** 1 Molecular Pharmacology Program, Sloan Kettering Institute, Memorial Sloan Kettering Cancer Center, New York, New York, USA.; 2 Pharmacology Department and; 3 Physiology Biophysics and Systems Biology Program, Weill Cornell Medicine, New York, New York, USA.

**Keywords:** Immunology, Antigen presentation, Immunotherapy, MHC class 1

## Abstract

Identification of MHC class I–bound peptides by immunopurification of MHC complexes and subsequent analysis by mass spectrometry is crucial for understanding T cell immunology and immunotherapy. Investigation of the steps for the MHC ligand isolation process revealed biases in widely used isolation techniques toward peptides of lower hydrophobicity. As MHC ligand hydrophobicity correlates positively with immunogenicity, identification of more hydrophobic MHC ligands could potentially lead to more effective isolation of immunogenic peptides as targets for immunotherapies. We solved this problem by use of higher concentrations of acetonitrile for the separation of MHC ligands and their respective complexes. This increased overall MHC ligand identifications by 2-fold, increased detection of cancer germline antigen–derived peptides by 50%, and resulted in profound variations in isolation efficacy between different MHC alleles correlating with the hydrophobicity of their anchor residues. Overall, these insights enabled a more complete view of the immunopeptidome and overcame a systematic underrepresentation of these critical MHC ligands of high hydrophobicity.

## Abstract 2

An approach is identified to prevent bias in the immunopeptidome towards MHC ligands of lower hydrophobicity and therefore immunogenicity.

## Graphical abstract

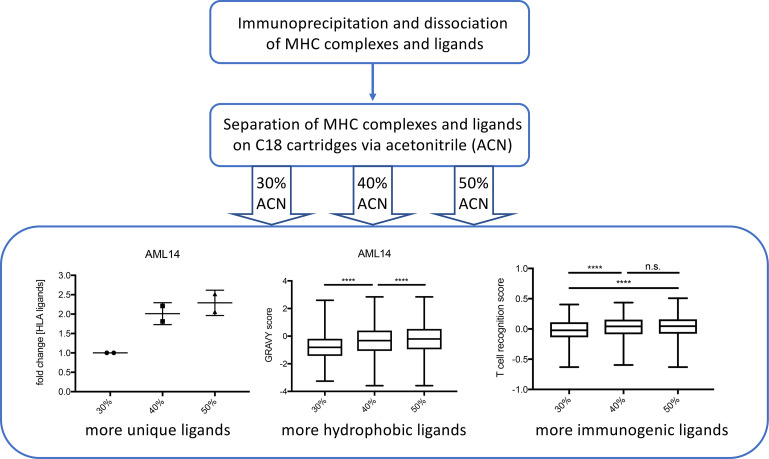

## Introduction

Defining the landscape of peptides presented on major histocompatibility complex (MHC) proteins provides better understanding of T cell immunity and supports the identification of immunotherapy targets (1–3). Several peptide isolation techniques have been standardized, but not benchmarked, resulting in various distinct experimental approaches (4–7). Though direct comparisons of these techniques remain sparse (8, 9), existing data point toward differences in the subsets of isolated MHC ligands depending on the type of protocol. Additionally, we hypothesized that anchor amino acid characteristics in distinct MHC alleles could influence isolation of the bound peptides in an allele-specific and method-dependent manner.

Therefore, we investigated several aspects of the isolation process to identify potential biases introduced by the biochemical characteristics of these approaches. Separation of the MHC ligand and complex appeared to be the dominant step yielding ligandome variations, which were usually skewed toward an underrepresentation of MHC ligands of higher hydrophobicity. Using hydrophobicity-dependent isolation via C18 cartridges in conjunction with increased concentrations of acetonitrile (ACN) enabled a more comprehensive collection of MHC ligands and their complexes; the number of unique peptide isolations was increased by about 2-fold with substantial enrichment for more hydrophobic peptides. Furthermore, the largest increases in identified MHC ligands were seen for MHC alleles with anchor site preferences for highly hydrophobic amino acids, e.g., HLA-A*02. Therefore, new methods might restore a previous imbalance within peptide isolation approaches and provide a more useful representation of the ligandome.

Finally, hydrophobicity of MHC ligands correlates positively with their immunogenicity (10). We corroborated these data through an HLA-A*02–specific reanalysis of a published data set, which led to the assumption that the number of potentially immunogenic epitopes might be elevated in our new MHC ligand subsets with higher hydrophobicity. This hypothesis was further supported by higher predictions of immunogenicity through the T cell recognition score by Calis et al. (11) when 9-mer HLA-A*02 ligands identified through different ACN elution conditions were investigated. Furthermore, our analysis identified 76 cancer germline antigen–derived (CGA-derived) peptides from 3 cell lines. Eleven of these HLA ligands have not been described before to our knowledge, including 2 binders from the well-studied immunogenic CGAs, cyclin A1 (12, 13), and MAGE-A12 (14, 15); the latter peptide was identified only in the group with the highest concentration of ACN.

Overall, this study discovered a bias in the known immunopeptidome that favors more hydrophilic and possibly less immunogenic peptides. Resolution of the problem by more stringent biochemical isolation conditions could have broad implications for the fields of immunology and immunotherapy because it has the potential to redefine and enlarge the repertoire of identified MHC ligands and to deepen the understanding of the immunopeptidome.

## Results

### Separation of MHC ligands and complexes is the most influential step for MHC ligand isolation and highly protocol dependent.

As many MHC isolation strategies differ widely, we compared method variations using 50 million BV173 cells per condition. Alterations were made at (a) the level of cell lysis, (b) antibody coupling/column preparation, and (c) the separation of MHC ligand and complex (Supplemental Figure 1A; supplemental material available online with this article; https://doi.org/10.1172/jci.insight.141264DS1). No significant differences were seen between the use of the detergent 3-([3-cholamidopropyl] dimethylammonio)-1-propanesulfonate (CHAPS) and the combination of octyl-β-d-glucopyranoside and sodium desoxycholate (OGP/SDC), although there was a trend for superior performance of CHAPS (Supplemental Figure 1B and Supplemental Table 1).

Similarly, no major changes in peptide yields were observed for antibodies either bound or cross-linked to protein A–Sepharose beads or for covalent coupling of antibodies to a cyanogen bromide–activated Sepharose 4B (Supplemental Figure 1C and Supplemental Table 1). A clear dose-dependent correlation was seen for the amount of antibody used (*R*^2^ = 0.994), allowing an optimal recovery of unique MHC ligands when at least a total of 0.5 mg of W6/32 antibody was used (Supplemental Figure 1D and Supplemental Table 1). All subsequent experiments were therefore performed with 0.5 mg of W6/32 antibody.

**Figure 1:**
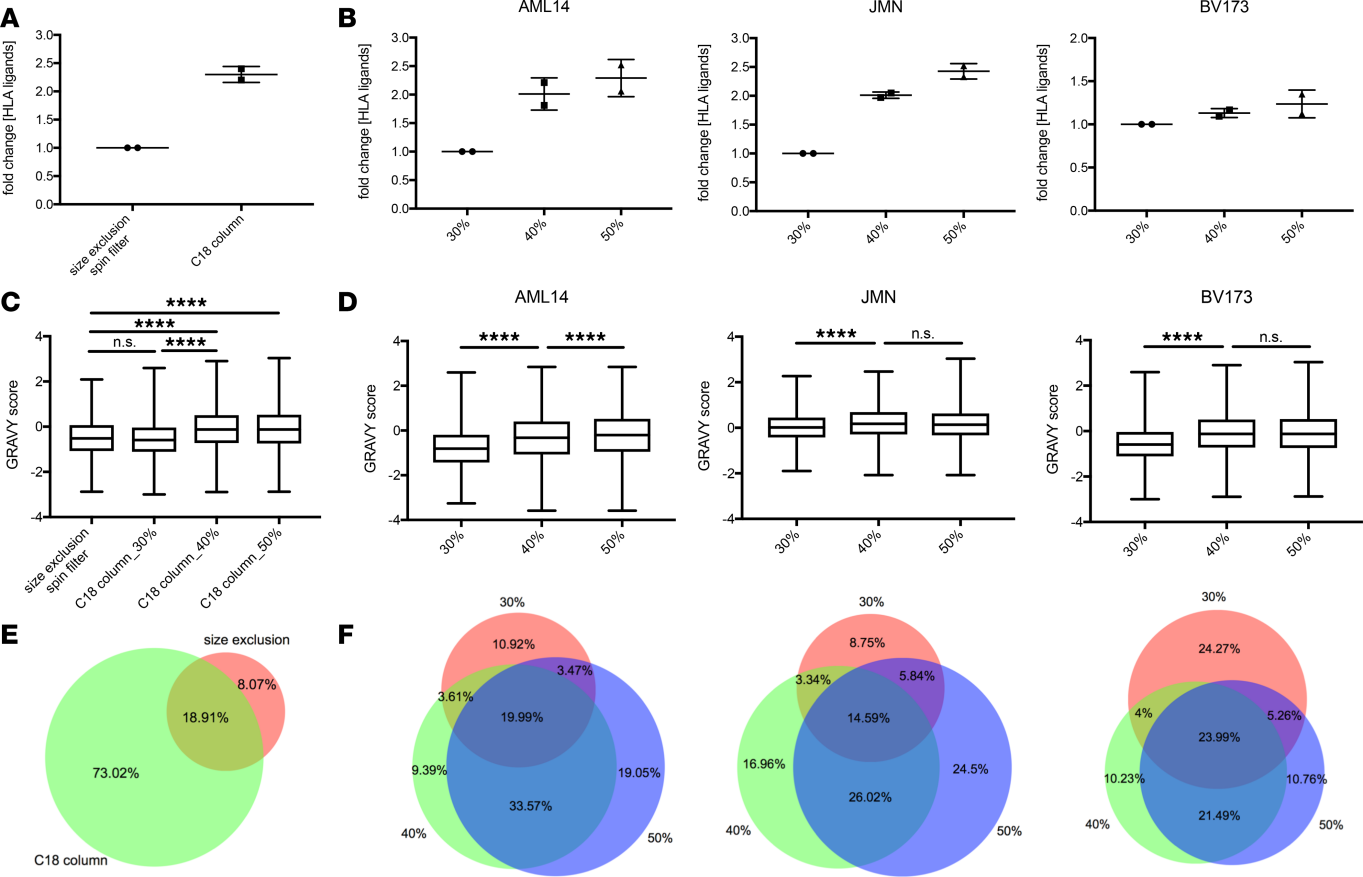
Characterization of MHC ligands eluted from C18 cartridges with various concentrations of ACN. (**A**) Comparison of size-exclusion spin filters and C18 cartridges with 30% ACN elution. (**B**) Relative changes for the yields of unique HLA ligands between different ACN elution conditions in AML14, JMN, and BV173 cells. (**C**) GRAVY scores for MHC-assigned peptides in BV173 cells. (**D**) GRAVY scores of different ACN elution conditions in AML14, JMN, and BV173 cells. (**E**) Venn diagram for size-exclusion spin filters and C18 cartridge experiments. Data sets were used from the experiment depicted in **C**. (**F**) Venn diagram for different ACN elution conditions in AML14, JMN, and BV173 cells. Data sets were used from experiments shown in **D**. Data were normalized to samples with lowest yield of unique HLA ligand identifications. Key: 30% ACN in red, 40% ACN in green, and 50% ACN in blue. For **A** and **B** mean with SD is indicated. In **C** and **D** whiskers show range of GRAVY scores from min to max. Boxes show mean with SD. One-way ANOVA test was used for multiple comparisons. ****P < 0.0001.

However, in the third step, separation of ligand and complex, we identified profound differences compared with the standard conditions. We compared 3 kDa size-exclusion spin filters (which segregate MHC peptide and complex by centrifugal force and only allow passage of the much smaller ligands whereas subunits of the MHC complex are retained) with C18 cartridges. These C18 columns bind the eluted MHC complexes and peptides that were previously dissociated by the use of 1% trifluoracetic acid (TFA) through hydrophobic interactions. By using polar reagents, such as a mixture of 30% ACN/0.1% TFA, MHC ligands are separated from the much more hydrophobic MHC complexes that remain bound to the C18 column. With C18 cartridge separation, we observed 2.5 times more unique MHC peptides compared with size-exclusion spin columns (Figure 1A). We assumed that the ACN elution was superior because of a more effective separation of more hydrophobic MHC ligands with stronger binding to the MHC binding groove. To test this hypothesis, we expanded this experimental approach by splitting immunopurified MHC complexes from AML14, JMN, or BV173 cells equally into 3 fractions; bound every fraction to separate C18 columns; and then eluted peptides with 30%, 40%, or 50% ACN. For all cell lines, the increased concentrations of ACN led to improved peptide recovery, with over 2-fold increases for AML14 and JMN and 30% for BV173 (Figure 1B). These data led to the hypothesis that these differences in recovery improvement may be related to the specific biochemical characteristics of the HLA alleles, which will be discussed later.

### Hydrophobicity of eluted MHC ligands correlates with concentrations of ACN used for MHC ligand isolation.

Because the use of higher concentrations of ACN led to a consistent increase in unique MHC ligand identifications, we next asked whether the chemical properties of the eluted ligands differed between isolation conditions. We used the grand average of hydropathicity index (GRAVY) (16) as a scale for hydrophobicity of a peptide, which is expected to increase when ligands are isolated with more polar reagents. Indeed, with higher concentrations of ACN, significant changes in the peptides’ average hydrophobicity were observed in BV173 cells (Figure 1C). In contrast, no significant difference in hydrophobicity was seen with MHC ligands isolated by the most commonly used isolation techniques (size exclusion or C18 cartridges eluted with 30% ACN).

Similar trends for the relationship between ACN and hydrophobicity were observed in AML14, JMN, and BV173 cells. In AML14 cells the increment from 40% to 50% ACN led to significant differences whereas for JMN and BV173 in the highest ACN groups, GRAVY scores were not statistically significant (Figure 1D). Importantly, every isolation condition led to a different set of MHC ligands, related to the method of isolation (Figure 1, E and F; and Supplemental Table 2). However, higher concentrations of ACN always gained more newly isolated unique HLA ligands than they lost (Supplemental Figure 2).

### Efficiency of MHC ligand isolation is MHC allele dependent.

**Figure 2:**
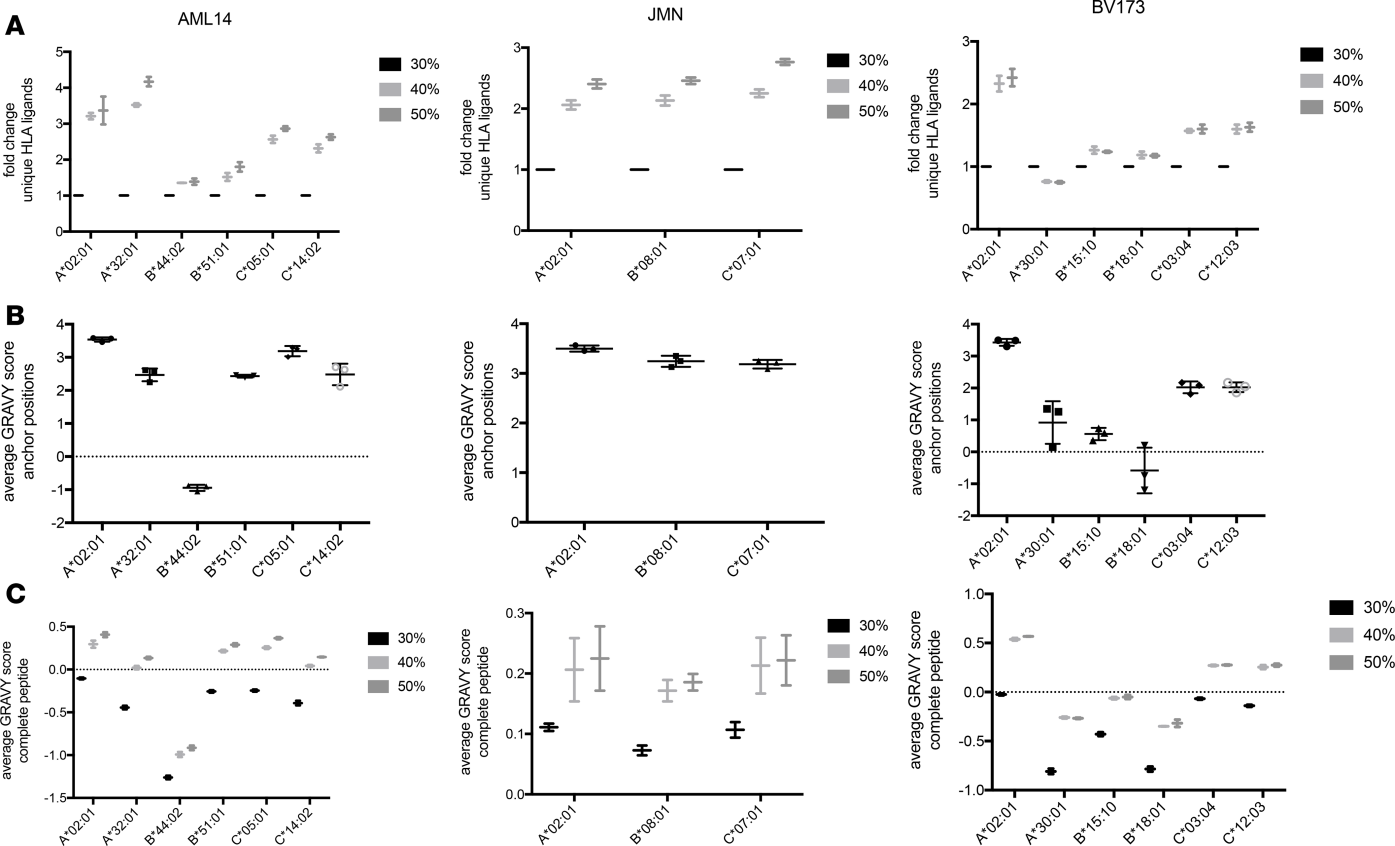
HLA allele–specific characterization of HLA ligands eluted from different ACN conditions. (**A**) Relative changes for the yields of unique HLA ligands between different ACN elution conditions and HLA alleles. HLA ligands were assigned to respective alleles by netMHCpan using a 2% rank cutoff. Results are normalized to the 30% ACN condition. (**B**) GRAVY scores for anchor amino acids (positions 2 and 9) in 9-mer HLA ligands. (**C**) GRAVY scores for complete 9-mer HLA ligands. In **A** and **C** whiskers indicate min to max. All experiments were performed in biological duplicates. In **B** mean with SD is indicated.

Because the hydrophobicity of anchor amino acids might influence the isolation process, we then asked whether the efficiency of ligand elution varied among MHC alleles. Such a result could further imply that the aforementioned bias in MHC ligand isolation could affect some MHC alleles and therefore some patient samples more than others. To test this hypothesis, we analyzed HLA assignments of ligands via netMHCpan 4.0 and normalized results to the 30% ACN specimens. While peptides increased with ACN concentrations for most alleles, HLA-A*30 showed a decrease by 25% in the total number of unique isolated MHC ligands with higher ACN (Figure 2A). The other MHC alleles demonstrated increases from 20% to over 400% in higher ACN conditions with high variation between individual alleles. Importantly, these profound differences in hydrophobicity and HLA allele–dependent increase in HLA ligands could not be achieved by the use of higher cell numbers, e.g., 200 million BV173 cells instead of 16.7 million, both eluted only with 30% ACN (Supplemental Figure 3 and Supplemental Table 2). The only MHC allele shared between the 3 studied cell lines, HLA-A*02:01, always showed at least a 2-fold increase in unique MHC ligand isolations. Because the most evident explanation for MHC allele dependency of this method would be a correlation with anchor amino acid characteristics, we calculated GRAVY scores for amino acids at positions 2 and 9 in all identified 9-mer MHC ligands for each allele, separately. Three major groups were identified that correlated with the average increase in MHC ligands per condition on the respective alleles: scores ranging from –1 to +1, scores from +1 to +3, and scores more than +3 (Figure 2B). For the first group with the most hydrophilic anchor residues (A*30:01, B*15:10, B*18:01, B*44:02), only small effects (±25%) were observed for alleles. The second subgroup (A*32:01, B*51:01, C*03,04, C*12:03, C*14:02) showed more varying effects, with increases between 50% (B*51:01) and 400% (A*32:01). More reliable trends were detected for the third group (A*02:01, B*08:01, C*05:01, C*07:01), with a constant 2- to 3-fold increase in unique MHC ligands between ACN subgroups.

To further determine the potential contribution of auxiliary anchors or other amino acid residues to the MHC allele–dependent isolation, we investigated the changes in the hydropathy of the complete peptide in an MHC allele– and ACN concentration–dependent manner. For all MHC alleles average hydrophobicity of eluted peptides went up with higher ACN concentrations, indicating that the effect was not solely attributable to anchor amino acid characteristics (Figure 2C).

### High concentrations of ACN for MHC ligand isolation might support identification of immunogenic MHC ligands and improve detection of CGA-derived MHC ligands.

**Figure 3:**
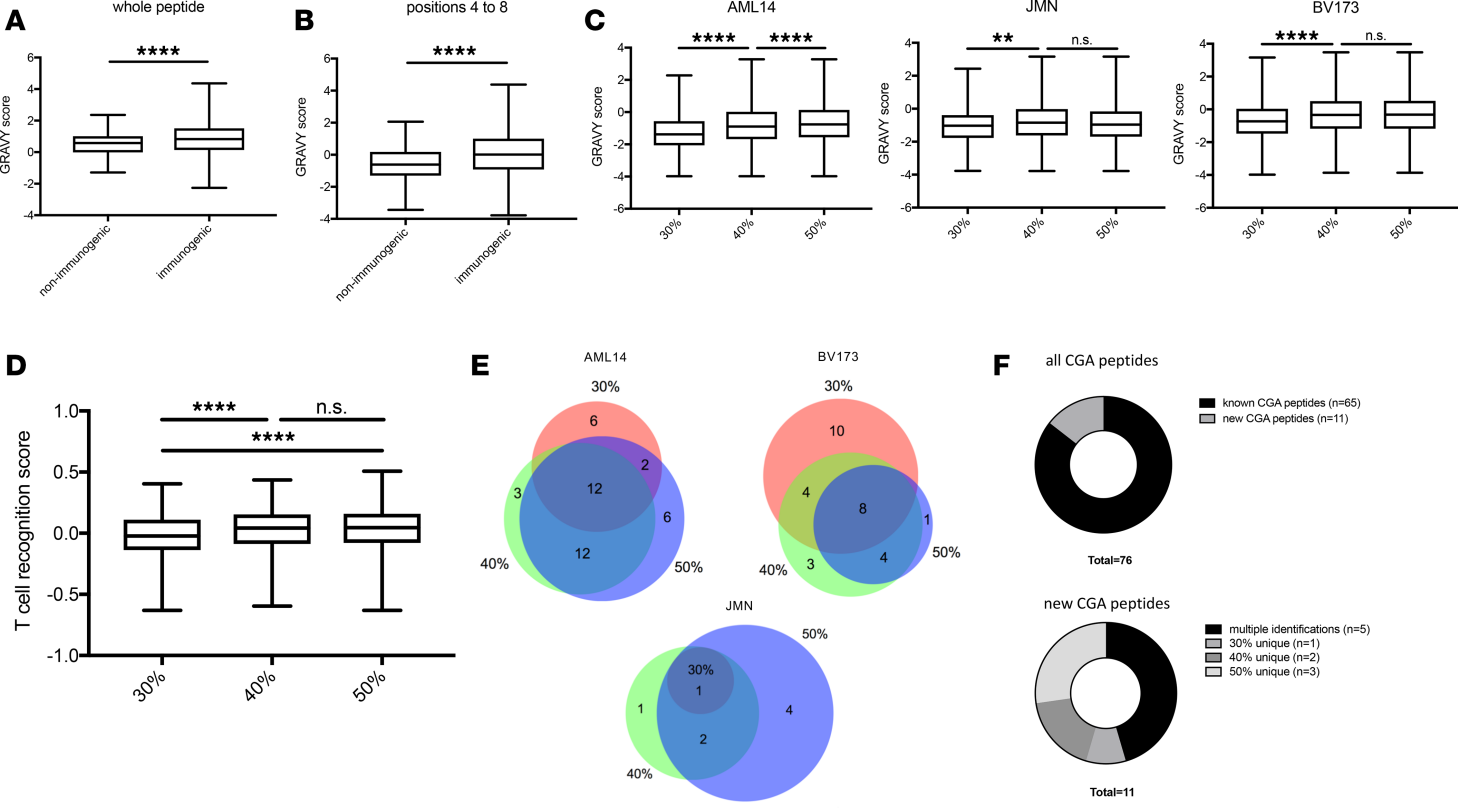
Correlation of hydrophobicity and immunogenicity. Epitopes eluted from CGAs. HLA-A*02 9-mer HLA ligands from Chowell et al. (10) were retrieved and GRAVY scores of immunogenic and nonimmunogenic peptides calculated for (**A**) the whole peptide and (**B**) amino acid positions 4 to 8. (**C**) GRAVY scores for positions 4 to 8 of 9-mer peptides identified in different ACN conditions. (**D**) T cell recognition score for 9-mer HLA-A*02 binder. (**E**) Venn diagrams for peptides derived from CGAs in different ACN elution conditions. Color key is the same as that for Figure 1. (**F**) Known and potentially novel peptides from CGAs. In **A**, **B**, **C**, and **D**, whiskers indicate min to max. Boxes show mean with SD. Experiments were performed in biological duplicates. One-way ANOVA test was used for multiple comparisons. **P < 0.01; ****P < 0.0001.

Next, we asked whether the observed higher hydrophobicity of the isolated MHC ligand could have implications for T cell recognition. On average, immunogenic MHC ligands display higher hydrophobicity compared with nonimmunogenic MHC-binding peptides (10) at their TCR recognition site. Therefore, we reanalyzed the provided data set by Chowell et al. focusing on HLA-A*02 binders. For HLA-A*02–binding 9-mer peptides, the GRAVY score was significantly higher in the immunogenic peptide group compared with the nonimmunogenic control group (Figure 3A). Even stronger differences in the average GRAVY were observed if the score was calculated only at the TCR recognition site (positions 4 to 8) (Figure 3B and Supplemental Table 3) (17). Interestingly, similar differences in GRAVY were observed for our cell lines among the various ACN concentrations (Figure 3C), especially between 30% ACN and higher concentrations.

We then asked if prediction algorithms for T cell recognition of MHC ligands can detect differences between the subgroups of peptides eluted in different ACN conditions. We used the T cell recognition score defined by Calis et al. (11) and included all 9-mer HLA-A*02 ligands detected in our study, because this algorithm is only validated for 9-mers and the most robust changes in the immunopeptidome were described for the HLA-A*02 allele. Strikingly, we observed significantly higher scores for the 40% and 50% ACN data sets compared with the 30% data set, further supporting our hypothesis that higher concentrations of ACN might support the isolation of more immunogenic HLA ligands (Figure 3D and Supplemental Table 3).

Finally, as another surrogate for immunogenicity of identified MHC ligands, we analyzed the detection of peptides from CGAs, as these antigens provide valuable targets for cancer immunotherapy (18, 19). We collected a list of 225 curated CGAs (Supplemental Table 3) (20) and matched these antigens to our data sets. For JMN, BV173, and AML14 cells, 8, 30, and 41 MHC ligands from CGAs were identified, respectively. Of importance, 88% (7/8), 27% (8/30), and 51% (21/41) of these subsets of peptides were exclusively found in the 40% and 50% ACN settings (Figure 3E). From a total of 76 CGA-derived detected peptides, 11 had not been described in the literature before. Moreover, 5 out of these 11 MHC ligands (45%) were only made detectable by use of 40% and 50% ACN in the elution conditions (Figure 3F), and 2 of the newly identified MHC ligands were derived from cyclin A1 (12, 13) and MAGE-A12 (14, 15), 2 antigens reported to be highly immunogenic. Though the MHC ligand from cyclin A1 could be detected in all 3 settings for the AML14 cell line, the MAGE-A12 peptide was identified only in a 50% ACN sample of JMN cells (Table 1).

## Discussion

Though various MHC isolation protocols are consistently used in the field, direct comparisons of these methods are scarce. We compared parameters of MHC ligand isolation, such as cell lysis conditions or antibody column preparation, and did not observe significant differences. CHAPS, however, was the detergent with the most favorable characteristics as consistent with the literature (9). Whereas one recent study showed higher yields with size-exclusion filters compared with C18 columns (9), in our experiments C18 cartridges eluted with ACN yielded 2.5 times more unique MHC ligands as compared with size-exclusion spin filters. To further investigate the effect of ACN on MHC ligand elutions, we increased concentrations of ACN up to 50% and detected even better recovery of MHC ligands in 3 cell lines, with over 2-fold increases in unique identifications. To determine the changes of abundance for specific HLA ligands between different ACN elution conditions in correlation to their GRAVY score, future studies might use stable isotope labeling of HLA ligands (21), which would allow a more precise characterization of changes in the immunopeptidome when using higher ACN concentrations for isolation of HLA ligands.

One study that used concentrations of ACN higher than 30% (22) pooled elutions of different ACN concentrations but did not investigate them separately. To reduce the risk for coelution of MHC complexes with the peptides when higher ACN concentrations were used, we performed solid-phase extractions before the sample was injected into the mass spectrometer (23).

With more unique MHC ligands identified in the samples of higher ACN concentrations, we hypothesized that the major differences in unique peptide yields can be attributed to the more hydrophobic properties of isolated MHC ligands. In contrast, for size-exclusion filters and C18 cartridges eluted with 30% ACN, the most commonly used strategies for separation of peptides and MHC complexes (4–7), no significant differences related to the hydrophobicity of identified peptides were detected. This implies that large subsets of peptides might be missed with standard isolation protocols and that the MHC ligands isolated within these 2 subgroups might be biased toward lower hydrophobicity. This indicated that for the most complete data sets of MHC ligand isolations, various ACN elution concentrations should be employed; e.g., C18 cartridges can be eluted sequentially with 30%, 40%, and 50% ACN, and elution fractions can either be analyzed separately or pooled for a single MS analysis. This idea is further supported by recent data using HPLCs with ACN gradients for peptide MHC separation, resulting in higher yields compared with C18 cartridges (9). Based on the results shown here, we believe that this HPLC approach could further benefit from gradients up to 40% or 50% ACN. The upper limit of ACN might be cell line dependent as observed for the 3 lines used in this study.

Moreover, MHC allele–dependent differences in hydropathy of eluted ligands have been described before (24) and are in line with the respective hydrophobicity of their anchor amino acids. Consistent with these results, we observed a correlation between the hydrophobicity of anchor amino acids and the total increase in unique MHC ligands per allele when using more stringent elution conditions. Importantly, for HLA-A*02 a 2-fold increase was observed in every cell line even if the number of total ligands did not increase dramatically. In contrast, the use of higher cell numbers did not lead to an increase of mean hydrophobicity to a similar extent as seen with 40% ACN or to an HLA allele–specific increase of HLA ligands. Because HLA-A*02 is the best characterized HLA complex in the field, these analyses highlighted the importance of the findings and suggested that the number and characteristics of HLA-A*02 binders might have been systematically underestimated by the field.

Furthermore, the presence of hydrophobic amino acids at the TCR recognition site is thought to be a hallmark of TCR recognition because of improved binding kinetics, including a reduced desolvation penalty (10, 25). Because our improved strategy led to the identification of MHC ligands of higher hydrophobicity, we further investigated their characteristics at the TCR recognition site, which supported the trend seen before with more hydrophobic peptide segments between positions 4 and 8 of a 9-mer peptide in the 40% and 50% ACN elution samples. This might imply that our approach leads to more MHC ligands and to the discovery of peptides with greater chance of immunogenicity. Further support for this hypothesis was provided by immunogenicity predictions of HLA-A*02 restricted 9-mer HLA ligands from different elution conditions, which showed a highly significant difference (Figure 3D) in T cell recognition score, indicating higher potential for immunogenicity with increasing concentrations of ACN.

Another positive aspect of our method is the improved recovery of CGA-derived HLA ligands. On average, 50% more peptides from CGAs were detected in the 40% and 50% ACN samples compared with the 30% ACN samples.

In this study we discovered the importance of improving separation conditions for MHC ligands from their complexes for mass spectrometry analysis of the immunopeptidome. Our data suggest that current isolation protocols do not sufficiently separate peptides from MHC complexes after they have been dissociated by TFA, especially for MHC alleles with anchor amino acids of high hydrophobicity, e.g., HLA-A*02, which leads to a significant bias in the published immunopeptidome. Resolving the problem by the use of more polar conditions to separate MHC ligands and complexes not only will allow a more complete characterization of the immunopeptidome but also allows the possibility of identification of MHC ligands of higher immunogenicity due to the positive correlation of MHC ligand hydrophobicity and immunogenicity.

## Methods

### Cell lines.

AML14 cells were a gift from Ross Levine (Memorial Sloan Kettering Cancer Center, New York, New York, USA), and JMN cells were maintained at Sloan Kettering Institute. BV173 cells were provided by H.J. Stauss (University College London, London, United Kingdom). All cell lines were HLA typed by the Department of Cellular Immunology at Memorial Sloan Kettering Cancer Center. All cell lines were maintained in RPMI medium and supplemented with 10% FBS and 2 mM glutamine.

### Immunopurification of HLA class I ligands.

For immunopurification several variations of different protocols were employed (5–8). Suspension cells were harvested through direct resuspension, and adherent cell lines were harvested after incubating 15 minutes with CellStripper solution (Corning, catalog 25056CI). Harvested cells were pelleted and washed 3 times in ice-cold sterile PBS (Media Preparation Core, Memorial Sloan Kettering Cancer Center). For all comparisons of protocols, 50 million BV173 cells were used per experiment. In 1 experiment, 200 million BV173 cells were used with a 30% ACN isolation strategy. For experiments comparing different concentrations of ACN, 50 million cells were used as well, but TFA eluates were later split into 3 equal portions. Then, cells were lysed in 7.5 mL of either 1% CHAPS (MilliporeSigma, catalog C3023) or 0.25% SDC (MilliporeSigma, catalog 30970) in combination with 1% OGP (MilliporeSigma, catalog O8001), all dissolved in PBS, supplemented with protease inhibitors (cOmplete, Roche, catalog 11836145001). The combination strategy was used only once for a direct comparison with CHAPS. All other experiments were exclusively performed with CHAPS. Cell lysis was performed for 1 hour at 4°C, lysates were spun down for 1 hour with 20,000*g* at 4°C, and supernatant fluids were isolated.

Affinity columns were prepared according to the protocols mentioned above in 3 variants.

For variant 1, 40 mg of cyanogen bromide–activated–Sepharose 4B (MilliporeSigma, catalog C9142) was activated with 1 mM hydrochloric acid (MilliporeSigma, catalog 320331) for 30 minutes. Subsequently, 0.5 mg (and for the experiments considering amount of antibody usage, 0 μg, 50 μg, 200 μg, 500 μg, or 1000 μg) of W6/32 antibody (Bio X Cell, catalog BE0079) were coupled to Sepharose in the presence of binding buffer (150 mM sodium chloride, 50 mM sodium bicarbonate, pH 8.3; sodium chloride: MilliporeSigma, catalog S9888; sodium bicarbonate: MilliporeSigma, catalog S6014) for at least 2 hours at room temperature. Sepharose was blocked for 1 hour with glycine (MilliporeSigma, catalog 410225) and washed 3 times with PBS.

For variant 2, 100 μg of protein A–Sepharose beads (Thermo Fisher Scientific catalog 15918014) were incubated with 0.5 mg of W6/32 antibody in PBS overnight at 4°C and then washed 3 times.

For variant 3, 100 μg of protein A–Sepharose beads were incubated with 0.5 mg of W6/32 antibody in 0.2 M sodium borate buffer (MilliporeSigma, catalog B3545) overnight at 4°C, then cross-linked with 20 mM dimethyl pimelimidate (MilliporeSigma, catalog 80490), and finally washed 3 times in PBS.

Supernatants of cell lysates were run over the different types of columns through peristaltic pumps (Pharmacia Biotech, Model P-1) with 1 mL/min flow rate overnight in a cold room. Affinity columns were washed with PBS for 30 minutes and water for 30 minutes, then run dry, and HLA complexes were subsequently eluted 5 times with 200 μL 1% TFA (MilliporeSigma, catalog 02031). For experiments where different settings for MHC ligand and complex isolation were investigated, the TFA eluates were pooled and then split in as many portions as settings were investigated (mostly 3 portions for the 30%, 40%, and 50% ACN settings, a fourth portion if spin filters were examined).

For separation of HLA ligands from their HLA complexes, C18 columns (Sep-Pak C18 1 cc Vac Cartridge, 50 mg sorbent per cartridge, 37–55 μm particle size, Waters, catalog WAT054955) were prewashed with 80% ACN (MilliporeSigma, catalog 34998) in 0.1% TFA and equilibrated with 2 washes of 0.1% TFA. Samples were loaded, washed again with 0.1% TFA, and eluted in 400 μL of 30%, 40%, or 50% ACN in 0.1% TFA. For separation by size-exclusion filters 0.5 mL 3 kDa–cutoff filters were used (Millipore Sigma, catalog UFC5003). Before use spin filters and tubes were prewashed with 1% TFA overnight to reduce polyethylene glycol content. Samples were then loaded into the filters and spun at 14,000*g* for 40 minutes. Flow-throughs were used for further analysis. Sample volume was reduced by vacuum centrifugation for mass spectrometry analysis.

### Solid-phase extractions.

In-house C18 minicolumns were prepared as follows: for solid-phase extraction of 1 sample, 2 small disks of C18 material (1 mm in diameter) were punched out from CDS Empore C18 disks (Thermo Fisher Scientific, catalog 13-110-018) and transferred to the bottom of a 200 μL Axygen pipette tip (Thermo Fisher Scientific, catalog 12639535). Columns were washed once with 100 μL 80% ACN/0.1% TFA and equilibrated 3 times with 100 μL 1% TFA. All fluids were run through the column by centrifugation in mini tabletop centrifuges, and eluates were collected in Eppendorf tubes. Then, dried samples were resuspended in 100 μL 1% TFA and loaded onto the columns, washed twice with 100 μL 1% TFA, run dry, and eluted with 50 μL 80% ACN/0.1% TFA. Again, sample volume was reduced by vacuum centrifugation.

### Liquid chromatography-tandem mass spectrometry analysis of HLA ligands.

Samples were analyzed by high-resolution/high-accuracy liquid chromatography-tandem mass spectrometry (LC-MS/MS) (Lumos Fusion, Thermo Fisher Scientific). Peptides were separated using direct loading onto a packed-in-emitter C18 column (75 μm ID/12 cm, 3 μm particles, Nikkyo Technos Co., Ltd.). The gradient was delivered at 300 nL/min increasing linearly from 2% buffer B (0.1% formic acid in 80% ACN)/98% buffer A (0.1% formic acid) to 30% buffer B/70% buffer A, over 70 minutes. MS and MS/MS were operated at resolutions of 60,000 and 30,000, respectively. Only charge states 1, 2, and 3 were allowed. The isolation window was chosen as 1.6 thomsons, and collision energy was set at 30%. For MS/MS, maximum injection time was 100 ms with an automatic gain control of 50,000.

### MS data processing.

MS data were processed using Byonic software (version 2.7.84, Protein Metrics) through a custom-built computer server equipped with 4 Intel Xeon E5-4620 8-core CPUs operating at 2.2 GHz and 512 GB physical memory (Exxact Corporation). Mass accuracy for MS1 was set to 6 ppm and to 20 ppm for MS2. Digestion specificity was defined as unspecific, and only precursors with charges 1, 2, and 3 and up to 2 kDa were allowed. Protein FDR was disabled to allow complete assessment of potential peptide identifications. Oxidization of methionine; phosphorylation of serine, threonine, and tyrosine; as well as N-terminal acetylation were set as variable modifications for all samples. Samples were searched against UniProt Human Reviewed database (20,349 entries, http://www.uniprot.org, downloaded June 2017) with common contaminants added. Peptides were selected with a minimal log probability value of 2, indicating *P* values for peptide spectrum matches of less than 0.01 and duplicates removed.

### Assignment of peptide sequences to HLA alleles.

To assign peptides that passed the MS quality filters described above to their HLA complexes that they most likely bind to, we used the netMHCpan 4.0 algorithm (24) with default settings. No binding affinity predictions were enabled. Therefore, all peptides with affinity percentage ranks below 2 were considered binders.

### Prediction of HLA ligand immunogenicity.

T cell recognition score was calculated for 9-mer HLA-A*02 binders using the online platform via IEDB (http://tools.iedb.org/immunogenicity/) (11).

### Statistics.

All graphs except Venn diagrams were drawn with GraphPad Prism 7. For statistics built-in analyses from GraphPad Prism were used. One-way ANOVA tests with Tukey’s multiple-comparisons test were used for comparing GRAVY scores in different isolation conditions. Venn diagrams were prepared using the BioVenn online platform (26). *P* values less than 0.05 were considered significant.

## Author contributions

MGK, KNM, YB, ZEHA, LIN, and SSM performed and analyzed experiments. MGK and DAS designed experiments. MGK wrote the original draft of the manuscript. TD and DAS supervised the project. DAS provided funding and edited the manuscript. All authors reviewed and contributed to the manuscript.

## Supplementary Material

Supplemental data

Supplemental Table 1

Supplemental Table 2

Supplemental Table 3

## Figures and Tables

**Table 1:**
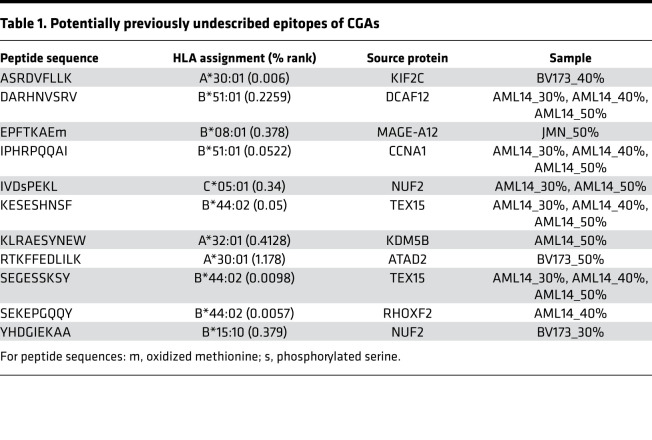
Potentially previously undescribed epitopes of CGAs

## Version 1 09/08/2020

In-Press Preview

## Version 2 10/02/2020

Electronic publication
