# Supplementary material for: Solving an MHC allele–specific bias in the reported immunopeptidome
Source: JCI Insight. 2020 Oct 2;5(19):e141264. doi: 10.1172/jci.insight.141264 (PMC7566711; doi:10.1172/jci.insight.141264)
Supplement: Supplemental data [file jciinsight-5-141264-s217.pdf]

Figure S1.

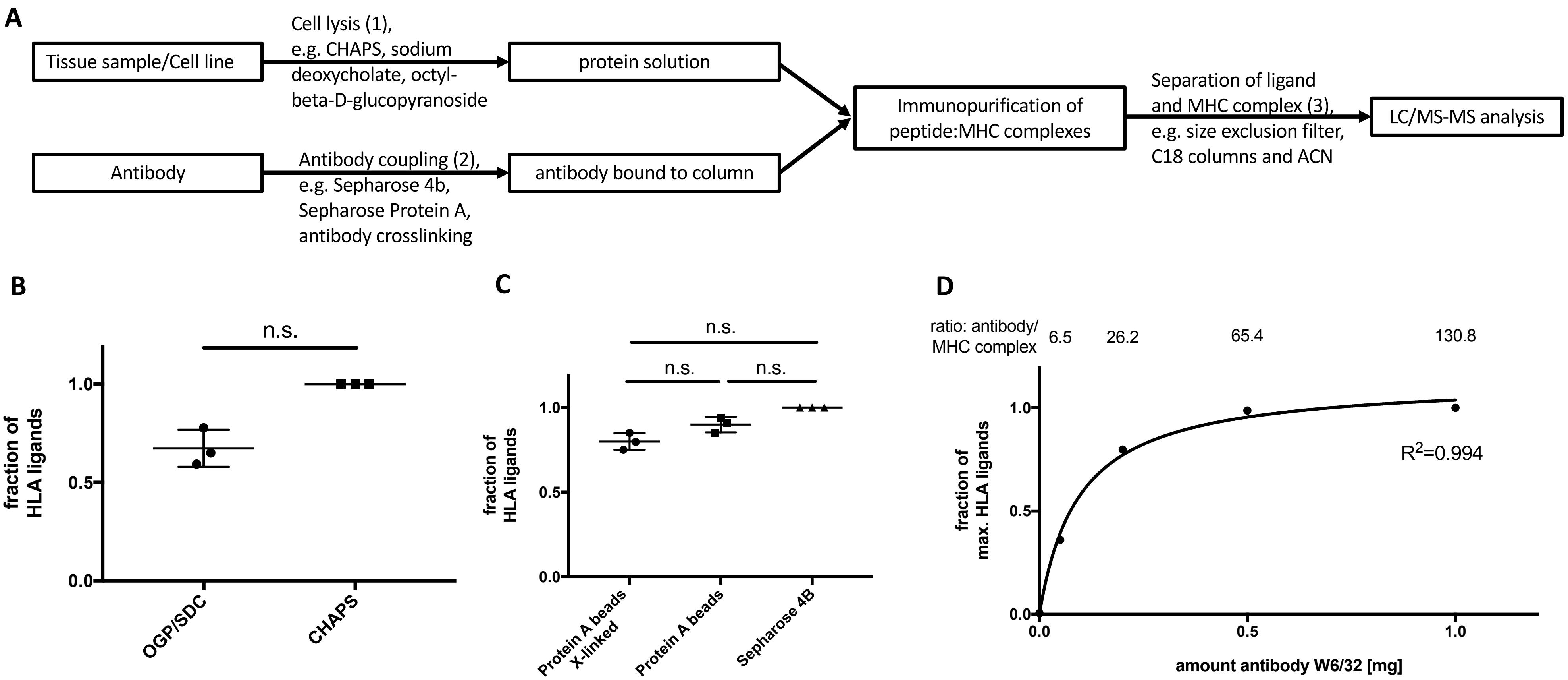

Fig. S1. Workflow and important protocol variants of immunopeptidome experiments. (A) Workflow of immunoprecipitation of an immunopeptidome experiment including most commonly used protocol variations. **(B)** Comparison of cell lysis conditions. **(C)** Comparison of antibody column preparation conditions. **(D)** Correlation of antibody input and unique HLA ligand identifications in experiment of 50M BV173 cells. All results are normalized to the condition with the highest yield. Error bars indicate mean with SD. For (B) and (C) experiments were performed in biological triplicates.

Figure S2.

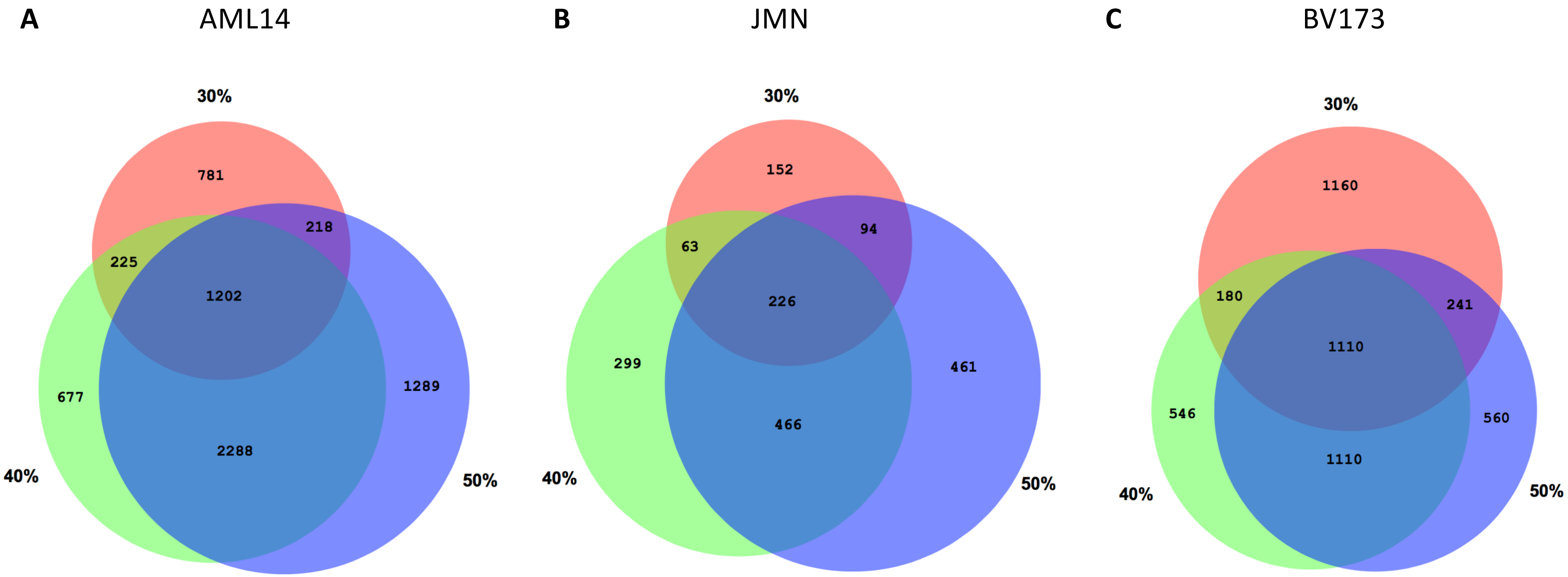

Fig. S2. Venn diagrams for absolute numbers of HLA ligands in different elution conditions. (A) AML14 cell **(B)** JMN cells **(C)** BV173 cells.

Figure S3.

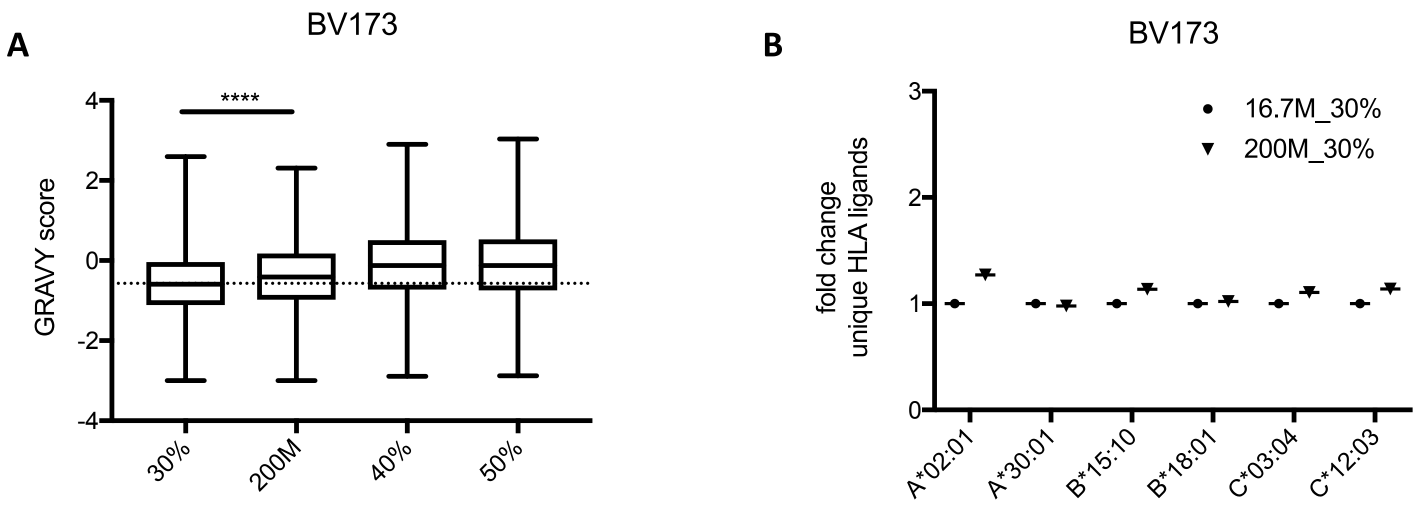

Fig. S3. Effects of increase in cell numbers on isolation characteristics. (A) GRAVY scores for different isolation conditions. 30%, 40% and 50% samples represent 16.7M BV173 cells as depicted in Fig. 1C. The new sample has a cell count of 200 M cells. **(B)** HLA allele-specific increase in unique HLA ligands Between 16.7M and 200M BV173 cells. Whiskers indicate min to max. Dotted line represents mean of 30% sample.
